# Neutral Lipids Are Not a Source of Arachidonic Acid for Lipid Mediator Signaling in Human Foamy Monocytes

**DOI:** 10.3390/cells8080941

**Published:** 2019-08-20

**Authors:** Carlos Guijas, Miguel A. Bermúdez, Clara Meana, Alma M. Astudillo, Laura Pereira, Lidia Fernández-Caballero, María A. Balboa, Jesús Balsinde

**Affiliations:** 1 Instituto de Biología y Genética Molecular, Consejo Superior de Investigaciones Científicas (CSIC), Universidad de Valladolid, 47003 Valladolid, Spain; 2 Centro de Investigación Biomédica en Red de Diabetes y Enfermedades Metabólicas Asociadas (CIBERDEM), 28029 Madrid, Spain

**Keywords:** arachidonic acid, mass spectrometry, lipid signaling, inflammation, phospholipase A_2_, monocytes/macrophages

## Abstract

Human monocytes exposed to free arachidonic acid (AA), a secretory product of endothelial cells, acquire a foamy phenotype which is due to the accumulation of cytoplasmic lipid droplets with high AA content. Recruitment of foamy monocytes to the inflamed endothelium contributes to the development of atherosclerotic lesions. In this work, we investigated the potential role of AA stored in the neutral lipids of foamy monocytes to be cleaved by lipases and contribute to lipid mediator signaling. To this end, we used mass spectrometry-based lipidomic approaches combined with strategies to generate monocytes with different concentrations of AA. Results from our experiments indicate that the phospholipid AA pool in monocytes is stable and does not change upon exposure of the cells to the external AA. On the contrary, the AA pool in triacylglycerol is expandable and can accommodate relatively large amounts of fatty acid. Stimulation of the cells with opsonized zymosan results in the expected decreases of cellular AA. Under all conditions examined, all of the AA decreases observed in stimulated cells were accounted for by decreases in the phospholipid pool; we failed to detect any contribution of the triacylglycerol pool to the response. Experiments utilizing selective inhibitors of phospholipid or triacylglyerol hydrolysis confirmed that the phospholipid pool is the sole contributor of the AA liberated by stimulated cells. Thus, the AA in the triacylglycerol is not a source of free AA for the lipid mediator signaling during stimulation of human foamy monocytes and may be used for other cellular functions.

## 1. Introduction

Mammalian cells store excess neutral lipids in cytoplasmic organelles called lipid droplets (LDs) [1]. LDs are present in practically all cell types, albeit their number and size may vary considerably from cell to cell. They are composed of a phospholipid monolayer decorated by a variety of proteins that encases a neutral lipid core consisting primarily of triacylglycerol (TAG) and cholesteryl esters (CEs). For many years, LDs were thought of only as mere storage depots for neutral lipids to be mobilized in case of energy needs. Today we know that, in addition to that storage function, lipid droplets serve a wide variety of roles in cell physiology [1]. Among these, LDs play prominent roles in the regulation of cell signaling by acting as platforms for signaling enzymes to dock and interact; this is particularly true for enzymes involved in lipid signaling; e.g., cytosolic phospholipase A_2_α (cPLA_2_α), cyclooxygenase-2 or lipin-1, all localize to this organelle [2,3]. LDs have also been found to play key roles in the development and progression of inflammatory metabolic disorders, of which the most prevalent is cardiovascular disease [4,5].

Atherosclerosis, a major cause of cardiovascular disease, is initiated by the abnormal activation of endothelial cells, which occurs under a variety of pathophysiological conditions such as e.g., increased blood levels of lipids (dyslipidemia) or sugar (diabetes). Endothelial cells release a variety of products with inflammatory potential that may attract monocytes and favor the interaction of these monocytes with the endothelial cells, resulting in the infiltration of activated monocytes into the vessel wall. There, the monocytes differentiate into macrophages, take up large amounts of lipids that have deposited into the subendothelial space, and participate decisively in the development of the atheroma plaque [6,7,8].

Arachidonic acid (AA) is among the many compounds secreted by endothelial cells that may affect monocytes. Damaged endothelium secretes relatively large amounts of this fatty acid, with the capacity to activate monocytes [9]. We have recently shown that human monocytes exposed to concentrations of AA similar to those secreted locally by endothelial cells, i.e., around 10 µM [9,10], acquire a foamy phenotype which is due to the accumulation of cytoplasmic lipid droplets [11,12]. This is a significant finding because it shows that monocytes may become foamy cells well before they gain access to the subendothelial space, increasing the cardiovascular risk [13]. Thus, as foamy monocytes can be found in circulation [13,14], it is possible that some specific molecular marker or signature exists in these cells, whose identification and characterization could constitute a useful tool for early detection of cardiovascular disease [12].

Importantly, the accumulation of AA into the neutral lipids of foamy monocytes also raises the question of whether this may have immediate pathophysiological consequences during inflammation. Monocytes, as major phagocytic cells that migrate to sites of inflammation, are key producers of AA-derived eicosanoids by mechanisms thought to be regulated by phospholipase A_2_ enzymes acting on AA-containing membrane phospholipids [15,16,17]. Thus, if the AA accumulating in neutral lipids within LDs can also be mobilized during inflammation, it would constitute a hitherto uncharacterized, second pathway for AA mobilization, alternative to the ‘classical’ phospholipase A_2_-driven AA mobilization from phospholipids. In the current study, we have tested this intriguing possibility by using advanced mass spectrometry-based lipidomic approaches, and various strategies to generate human monocytes containing numerous LDs. Our results indicate that, even under conditions where TAG constitutes the major cellular store of AA, the bulk of this fatty acid that is mobilized under cellular stimulation arises largely, if not exclusively, from phospholipids. Thus, the AA in TAG is not utilized for lipid mediator generation during inflammatory activation of monocytes and may serve other cellular functions.

## 2. Materials and Methods

### 2.1. Reagents

The cell culture medium was from the Corning Life Sciences (New York, NY, USA). Organic solvents (Optima^®^ LC/MS grade) were from Fisher Scientific (Madrid, Spain). Lipid standards were from Avanti (Alabaster, AL, USA) or Cayman (Ann Arbor, MI, USA). AA was purchased from either Sigma-Aldrich (Madrid, Spain), Larodan (Solna, Sweden), or Cayman. SilicaGel thin-layer chromatography plates were from Macherey-Nagel (Düren, Germany). The cytosolic phospholipase A_2_α (cPLA_2_α) inhibitor pyrrophenone was synthesized and provided by Dr. Alfonso Pérez (Department of Organic Chemistry, University of Valladolid). Bromoenol lactone (BEL) was from Cayman. All other reagents were from Sigma-Aldrich.

### 2.2. Cell Isolation and Culture

Human monocytes were obtained from buffy coats of healthy volunteer donors provided by the Centro de Hemoterapia y Hemodonación de Castilla y León (Valladolid, Spain). Written informed consent was obtained from each donor. In brief, blood cells were diluted 1:1 with phosphate-buffered saline, layered over a cushion of Ficoll-Paque, and centrifuged at 750× *g* for 30 min. The mononuclear cellular layer was recovered and washed three times with phosphate-buffered saline, resuspended in a RPMI 1640 medium supplemented with 40 µg/mL gentamicin, and allowed to adhere in sterile dishes for 2 h at 37 °C in a humidified atmosphere of CO_2_/air (1:19). Nonadherent cells were removed by washing extensively with phosphate-buffered saline, and the remaining attached monocytes were used the following day [18,19].

All experiments were conducted in serum-free media. Opsonized zymosan was added to the cells at 1 mg/mL for the times indicated. Zymosan was prepared exactly as described [19,20,21,22]. Only zymosan batches that demonstrated no measurable endogenous phospholipase A_2_ activity, as measured by in vitro assay under different conditions [23,24,25,26], were used in this study. Cell protein content was quantified according to Bradford [27], using a commercial kit (BioRad Protein Assay).

### 2.3. Gas Chromatography/Mass Spectrometry (GC/MS) Analyses

Total lipids from approximately 10^7^ cells were extracted according to Bligh and Dyer [28], and the following internal standards were added: 10 nmol of 1,2-diheptadecanoyl-*sn*-glycero-3-phosphocholine, 10 nmol of 1,2,3-triheptadecanoylglycerol, and 30 nmol of cholesteryl tridecanoate. Phospholipids were separated from neutral lipids by thin-layer chromatography, using *n*-hexane/diethyl ether/acetic acid (70:30:1, v/v/v) as the mobile phase [29]. Phospholipid classes were separated twice with chloroform/methanol/28% (w/w) ammonium hydroxide (60:37.5:4, v/v/v) as the mobile phase, using plates impregnated with boric acid [30]. The bands corresponding to the different lipid classes were scraped off from the plate, and fatty acid methyl esters were obtained from the various lipid fractions by transmethylation with 0.5 M KOH in methanol for 60 min at 37 °C [11,12,31,32]. Analysis was carried out using an Agilent 7890A gas chromatograph coupled to an Agilent 5975C mass-selective detector operated in an electron impact mode (EI, 70 eV), equipped with an Agilent 7693 autosampler and an Agilent DB23 column (60 m length × 0.25 mm internal diameter × 0.15 µM film thickness) (Agilent Technologies, Santa Clara, CA, USA). Data analysis was carried out with the Agilent G1701EA MSD Productivity Chemstation software, revision E.02.00 [11,12,31,32].

### 2.4. Liquid Chromatography/Mass Spectrometry (LC/MS) Analyses of Phospholipids

Lipids were extracted according to Bligh and Dyer [28], and the following internal standards were added: 20 pmol each of 1,2-dimyristoyl-*sn*-glycero-3-phosphoglycerol, 1,2-dilauroyl-*sn*-glycero-3-phosphoethanolamine, 1,2-dimyristoyl-*sn*-glycero-3-phosphoethanolamine, 1,2-diheptadecanoyl-*sn*-glycero-3-phosphoethanolamine, 1,2-dimyristoyl-*sn*-glycero-3-phosphoserine, 1,2-dimyristoyl-*sn*-glycero-3-phosphate, 1,2-dipentadecanoyl-*sn*-glycero-3-phosphocholine, 1,2-diheptadecanoyl-*sn*-glycero-3-phosphocholine, and 1,2-dinonadecanoyl-*sn*-glycero-3-phosphocholine. The samples were re-dissolved in 50 μL of hexanes/2-propanol/water (42:56:2, v/v/v), and 40 μL was injected into an Agilent 1260 Infinity high-performance liquid chromatograph equipped with an Agilent G1311C quaternary pump and an Agilent G1329B autosampler (Agilent Technologies, Santa Clara, CA, USA). The column was a FORTIS HILIC (150 × 3 mm, 3 μm particle size) (Fortis Technologies, Geston, UK), protected with a Supelguard LC-Si (20 mm × 2.1 mm) cartridge (Sigma-Aldrich, Madrid, Spain). The mobile phase consisted of a gradient of solvent A (hexanes/2-propanol, 30:40, v/v) and solvent B (hexanes/2-propanol/20 mM ammonium acetate in water, 30:40:7, v/v/v). The gradient started at 75% A from zero to 5 min, then decreased from 75% A to 40% A at 15 min, from 40% A to 5% A at 20 min, holding at 5% until 40 min, and increasing to 75% at 41 min. The column was then re-equilibrated by holding at 75% A for an additional 14 min before the next sample injection. The flow rate through the column was fixed at 400 μL/min, and this flow entered into the electrospray ionization interface of a Sciex QTRAP 4500 hybrid triple quadropole mass spectrometer operated in a negative ion mode (AB Sciex, Framingham, MA, USA). Source parameters were as follows: ion spray voltage, −4500 V; curtain gas, 30 psi; nebulizer gas, 50 psi; desolvation gas, 60 psi; temperature, 425 °C. Phospholipid species were detected as [M−H]^−^ ions except for choline phospholipids, which were detected as [M + CH_3_COO^−^]^−^ adducts, and were identified by comparison with previously published data [33,34,35,36,37,38,39,40].

### 2.5. Liquid Chromatography/Mass Spectrometry (LC/MS) Analyses of Arachidonic Acid

A volume of 90 µL of the sample diluted in eluent A was injected into an Agilent 1260 Infinity high-performance liquid chromatograph equipped with an Agilent G1311C quaternary pump and an Agilent G1329B autosampler. The column was a SUPELCOSIL LC-18 (250 × 2.1 mm, 5 µM particle size) protected with a Supelguard LC-18 (20 × 2.1 mm) guard cartridge (Sigma-Aldrich). Oxidized fractions were separated according to the procedure described by Dumlao et al. [41], with minor modifications. Briefly, the mobile phase consisted of a gradient of solvent A (water/acetonitrile/acetic acid, 70:30:0.02, by volume) and solvent B (acetonitrile/isopropanol, 50:50, by volume). The gradient was started at 100% of solvent A, which was decreased linearly to 75% at 3 min, 55% at 11 min, 40% at 13 min, 25% at 18 min, and 10% at 18.5 min. The last solvent mixture was held for an additional 1.5 min; finally, the column was re-equilibrated with 100% of solvent A for 10 min before the next sample injection. The flow rate through the column was fixed at 0.6 mL/min, and this flow entered into the electrospray ionization interface of a Sciex QTRAP 4500 hybrid triple quadropole mass spectrometer operated in negative ion mode (AB Sciex, Framingham, MA, USA). The parameters of the source were set as follows: Ion spray voltage, −4500 V; curtain gas, 30 psi; nebulizer gas, 50 psi; desolvation gas, 60 psi; temperature, 550 °C. Oxidized and non-oxidized molecules were analyzed as [M−H]^−^ ions.

### 2.6. LD Analysis by Confocal Microscopy

The LD content was examined with a Leica TCS SP5X confocal microscope (Wetzlar, Germany), using an oil immersion, 63×, 1.4 NA, HCX PL APO CS objective. The fluorescence from BODIPY 498/503 was monitored with a white light laser exciting at 490 nm, and fluorescence emission was collected between 502 nm and 555 nm. The DAPI fluoresce was excited with a blue diode laser at 405 nm and emission was collected between 439 nm and 4787 nm. Fluorescence quantification of no less than 150 cells per condition was analysed using the ImageJ software (version 1.52a).

### 2.7. Statistical Analysis

All experiments were carried out at least three times with incubations in duplicate or triplicate. Statistical analysis was carried out by the Student’s t test, with *p* < 0.05 taken as statistically significant.

## 3. Results

**Figure 1:**
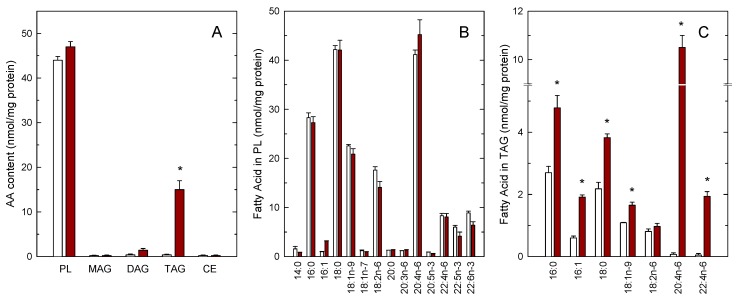
Arachidonic acid (AA) incorporation into the lipids of human monocytes. Monocytes were either untreated (open bars) or treated with 10 µM AA for 2 h (maroon bars). Afterwards, the various lipid classes were isolated and their AA content was measured by GC/MS after converting the fatty acid glyceryl esters into fatty acid methyl esters (**A**), the full profile of fatty acids of phospholipids (**B**) and triacylglycerol (TAG) (**C**) is also shown. Fatty acids are designated by their number of carbon atoms and, after a colon, their number of double bonds. To differentiate isomers, the *n*−x (*n* minus x) nomenclature is used, where *n* is the number of carbons of a given fatty acid, and x is an integer which, subtracted from *n* gives the position of the last double bond of the molecule. Results are shown as means ± SEM. (*n* = 3). * *p* < 0.05, significantly different from AA-untreated cells.

We have previously shown that incubating human monocytes with low micromolar concentrations of AA results in the cells acquiring a foamy-type phenotype as a consequence of the accumulation of cytoplasmic LDs [11,12]. In this study, we have utilized mass-spectrometry based lipidomic approaches to characterize further the lipid composition of these foamy monocytes with regard to the AA distribution in the various lipid classes. In resting, untreated monocytes, endogenous AA was present almost exclusively in the phospholipid fraction; the AA content of all neutral lipid fractions (monoacylglycerol, MAG; diacylglycerol, DAG; triacylglycerol, TAG; cholesteryl esters, CE) was negligible (Figure 1A). Interestingly, when the cells were exposed to 10 µM AA for 2 h, the TAG fraction constituted the only cellular lipid class that incorporated significant amounts of AA (Figure 1A). The phospholipid (PL) fraction incorporated only small amounts of AA, suggesting that this pool is almost replete in the resting monocyte and cannot significantly accommodate more AA [17,42]. Full fatty acid distribution analyses by GC/MS confirmed small, not significant changes of AA in the phospholipid fraction. Upon AA supplementation, we also noted small decreases in the linoleic acid (18:2n–6) and the polyunsaturates of the n–3 series (22:5n–3 and 22:6n–3), suggesting that these are the fatty acids that are likely displaced within phospholipids by the small amounts of AA entering the phospholipid fraction. However, none of these differences reached statistical significance (Figure 1B). Interestingly, the GC/MS analyses of fatty acids in TAG revealed not only the expected increases in AA and its elongation product adrenic acid (22:4n–6), but also of palmitic acid (16:0), palmitoleic acid (16:1), stearic acid (18:0) and oleic acid (18:1n–9), confirming that the exogenous AA also activates de novo fatty acid synthesis in monocytes, in agreement with previous estimates [11].

**Figure 2:**
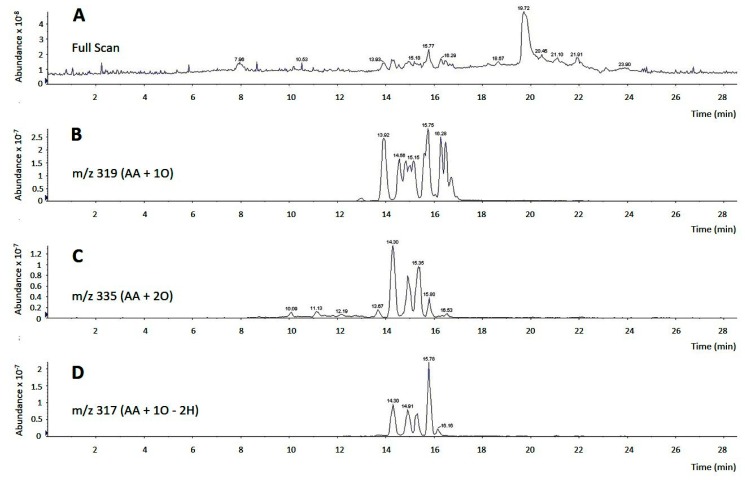
LC/MS analysis of commercial AA. (**A**) Full scan chromatogram of a commercial AA sample, showing a major peak at 19.72 min, representing pure, non-oxidized AA, and groups of peaks between 13.93 min and 16.53 min, representing multiple oxidized AA derivatives; (**B**–**D**) analyses of oxidized derivatives of commercial AA. In panel B, m/z 319 was selected to detect arachidonate hydroxides and epoxides (m/z of AA plus one oxygen atom). In panel C, m/z 335 was selected to detect arachidonate hydroperoxides (m/z of AA plus two oxygen atoms). In panel D, m/z 317 was selected to detect arachidonate derivatives containing an oxo group (m/z of AA plus one oxygen atom minus two hydrogen atoms).

In our previous work we had noted that the biological effects of AA on monocytes were not affected by the presence of cyclooxygenase or lipoxygenase inhibitors [11], thus suggesting that they were due to the fatty acid itself and not to an oxygenated metabolite produced by the cells after they came in contact with the AA. However, commercial samples of AA frequently contain decided amounts of oxidized products, which raises the concern of whether these compounds could possess biological activity on their own which obscure that of the pure free fatty acid. To test this possibility, we analyzed by LC/MS the composition of commercial AA samples acquired from three different suppliers. Depending on the supplier and shipping conditions (neat or dissolved in organic solvents; presence or not of an anti-oxidant), we found that between 5–35% of product, immediately opened upon arrival in the lab, was present in the form of oxidized compounds containing hydroxy, hydroperoxy or oxo groups (Figure 2). LC/MS/MS analyses in product-ion scan mode utilizing specific fragments, comparison with bibliographic data [43,44] and, when available, comparison of retention times and fragments with appropriate standards, allowed identification of most of the oxidized products present in the samples (Table 1).

**Figure 3:**
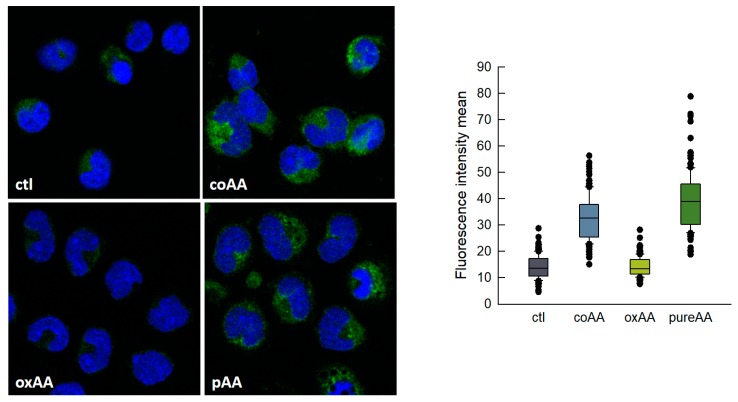
AA-induced lipid droplets (LDs) formation in human monocytes. Monocytes were untreated (ctl) or treated with commercial AA (coAA), oxidized AA (oxAA) or pure AA (pAA) for 2 h, as indicated. In all cases, the fatty acid was added at a concentration of 10 μM. After fixation and permeabilization, cells were stained with boron-dipyrromethene (BODIPY) 493/503 (2 µg/mL) to visualize LD (green) and 4′,6′-diamidino-2-phenylindole (DAPI) (1 µg/mL) to mark the nuclei (blue). Cells were then analyzed by confocal microscopy (original magnification 630×). The boxplot on the right shows the distribution of the fluorescence intensity data based on a summary of median, minimum, maximum and quartiles from at least 150 cells in two independent experiments.

Non-oxidized AA could be easily separated from its oxidized derivatives by thin-layer chromatography using a system consisting of *n*-hexane/ethyl ether/acetic acid (70:30:1) (Rf of pure AA, 0.66; Rf of oxidized products, 0.27–0.46). Thus we isolated pure, non-oxidized AA and re-analyzed its effect on LD formation by human monocytes. We also analyzed the effect of oxidized AA fractions. The results are shown in Figure 3, and clearly indicate that it is indeed pure AA that is responsible for the cells to acquire a foamy phenotype. Oxidized AA fractions had little or no effect on the response. There was no difference in the uptake of oxidized and non-oxidized fatty acid by the cells. The uptake was calculated by measuring the amount of fatty acid remaining in the media at the end of the incubations [45,46].

**Figure 4:**
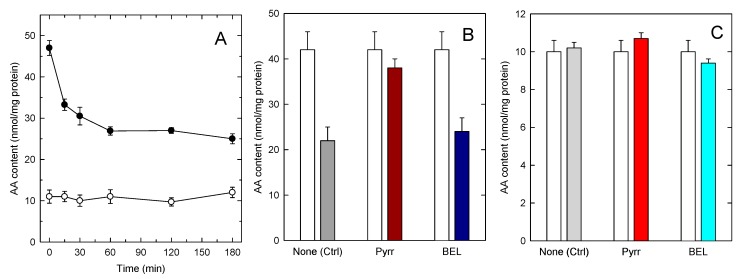
AA mobilization by zymosan-stimulated human monocytes. (**A**) The cells were stimulated with 1 mg/mL opsonized zymosan for the indicated times. Afterwards, the AA content in phospholipids (black circles) or TAG (open circles) was analyzed by GC/MS; (**B**,**C**) the cells were preincubated with either 1 µM pyrrophenone, 10 µM BEL or neither (none) for 30 min, as indicated. Afterwards, the cells were either untreated (open bars) or treated with 1 mg/mL opsonized zymosan for 2 h. The AA content in phospholipids (**B**) or TAG (**C**) was analyzed by GC/MS. Data are expressed as means ± SEM of three independent determinations.

The increased accumulation of AA in TAG in foamy monocytes is particularly important from a pathophysiological point of view because foamy monocytes are present at sites of inflammation. Thus, it was of interest to investigate whether the AA present in TAG can be mobilized upon cellular activation. To begin to address this question, we set to characterize AA mobilization in activated monocytes by mass spectrometry. In the first series of experiments, we analyzed AA mobilization from foamy monocytes in response to a well defined proinflammatory stimulus, opsonized zymosan [47]. Figure 4A shows that opsonized zymosan did stimulate abundant AA mobilization from foamy monocytes; however GC/MS analyses of the AA content of phospholipid and TAG fractions clearly indicated that the origin of the released fatty acid was primarily, if not exclusively, the phospholipid fraction. To further substantiate these findings, experiments were carried out with inhibitors of the enzymes putatively involved in effecting the AA release from either phospholipids, namely cytosolic group IVA phospholipase A_2_ (cPLA_2_α), or TAG, namely TAG lipase (ATGL). To inhibit cPLA_2_α we used the well-established inhibitor pyrrophenone [48,49,50], and to block ATGL we used bromoenol lactone (BEL), a general inhibitor of patatin-like phospholipases, that has been shown to efficiently prevent ATGL-induced TAG hydrolysis [51,52,53]. Figure 4B,C show that while pyrrophenone almost completely prevented the AA loss from the stimulated monocytes, BEL had no appreciable effect. Note in Figure 4B,C that BEL did not affect the AA mobilization from phospholipids, and that pyrrophenone did not affect the AA levels in TAG, reflecting the specificity of action of both compounds.

**Figure 5:**
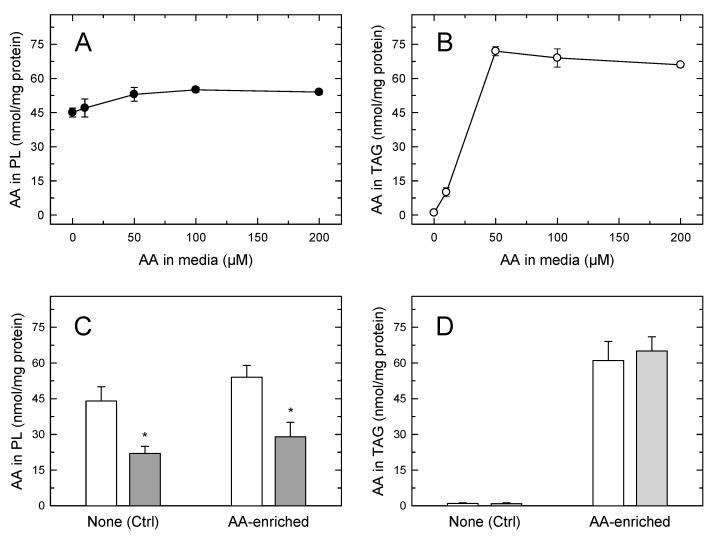
The AA content of human monocytes incubated in AA-rich media (**A**,**B**). The cells were incubated with the indicated concentration of AA for 24 h. The fatty acid was added to the media complexed with bovine serum albumin at a 2:1 ratio. After the incubations, the AA content of cellular phospholipids (**A**) or TAG (**B**) was analyzed by GC/MS. Comparison of the AA mobilization responses between normal circulating cells and AA-enriched cells (**C**,**D**); the cells were either untreated (open) bars) or treated with 1 mg/mL opsonized zymosan for 2 h (grey bars). The AA content in phospholipids (**C**) or TAG (**D**) was analyzed by GC/MS. Data are expressed as means ± SEM of three independent determinations. * *p* < 0.05, significantly different from unstimulated cells.

In the experiments depicted in Figure 4 it is noted that, despite the foamy phenotype of the monocytes, the amount of AA in phospholipids still clearly exceeded that of the fatty acid in TAG. This prompts the question of whether the AA mobilization would be different in cells where TAG, not phospholipid, constitutes the major cellular AA reservoir. To address this question, we incubated the monocytes with increasing concentrations of AA (up to 200 µM) for 24 h, so that incorporation was only limited by the intrinsic capacity of the cells to expand their lipid pools to accommodate the fatty acid [17,42]. Since AA concentrations ≥ 50 µM are cytotoxic to most cell types [54,55,56,57], for these experiments the fatty acid was complexed with bovine serum albumin (2:1 ratio) before adding it to the cells. In addition to providing a more physiological setting for the cells to incorporate the fatty acid, this procedure preserved cell viability above 98% along the course of the incubations. At low non-toxic concentrations, the AA incorporation into cellular lipids is the same whether or not the fatty acid is complexed with albumin. Figure 5A shows that the amount of AA in phospholipid changed very little even after incubating the cells with a 200 µM fatty acid, indicating again that circulating monocytes already contain in phospholipids practically all the AA they can accommodate; thus the phospholipid pool is not expandable further. Conversely, incubation of the cells with ≥50 µM of AA for 24 h resulted in the TAG fraction incorporating AA to levels well above those found in phospholipids (Figure 5B). Thus, by expanding their TAG pool, monocytes have the capacity to increase their cellular AA content by 2–2.5-fold compared to that found in circulating cells. Stimulation of the AA-enriched monocytes with opsonized zymosan resulted in the expected sharp decreases in the cellular AA content (Figure 5C,D). The AA mobilization response was similar in magnitude to that experienced by cells not enriched with AA, and only phospholipids, not TAG, contributed to it (Figure 5C,D). Thus, even in cells containing more AA in TAG than in phospholipids, receptor-stimulated AA mobilization is due to the hydrolysis of phospholipids, not of TAG.

**Figure 6:**
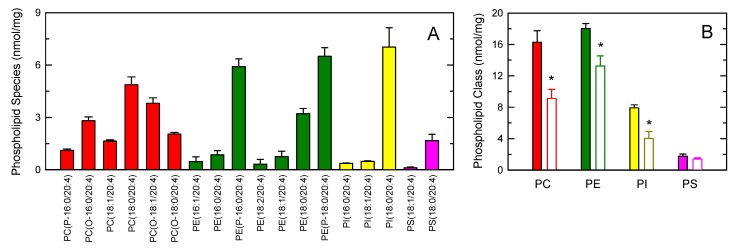
AA-containing phospholipid species in human monocytes. (**A**) The distribution profile of AA between choline-containing phospholipids (PC, red bars), ethanolamine-containing phospholipids (PE, green bars), phosphatidylinositol (PI, yellow bars), and phosphatidylserine (PS, pink bars) species was determined by LC/MS/MS; (**B**) AA content in phospholipids as shown by the class and effect of the cellular stimulation. The cells were not stimulated (colored bars) or stimulated by 1 mg/mL opsonized zymosan for 2 h (open bars). Afterward, the different phospholipid classes were separated by thin-layer chromatography and their AA content was determined by GC/MS after converting the fatty acid glyceryl esters into fatty acid methyl esters. Data are expressed as means ± SEM of three independent determinations. * *p* < 0.05, significantly different from unstimulated cells.

Figure 6A shows the distribution profile of AA between phospholipids, as measured by LC/MS/MS. Multiple AA-containing species were detected within the major phospholipid classes, with ethanolamine plasmalogens and the inositol species PI(18:0/20:4) containing the highest amounts of the fatty acid. At the class level, PC and PE were found to contain similar amounts of AA, while PI and PS contained lower levels (Figure 6B). Stimulation of the cells with opsonized zymosan resulted in marked decreases of AA in PC, PE and PI. Note that, during cell activation, CoA-independent transacylase reactions replenish the AA pool in PE (both 1-acyl and 1-alkenyl species) with fatty acid originating from PC (1-acyl species) [35,38,42,58,59,60]; thus, the actual contribution of PE to the overall AA release is likely higher than that reflected in Figure 6B.

## 4. Discussion

Innate immune cells such as circulating blood monocytes contain large amounts of AA which can be mobilized under a variety of pathophysiological conditions [9]. The fatty acid can be metabolized into a relatively high number of oxygenated metabolites, collectively called the eicosanoids, by four major routes, namely cyclooxygenase, lipoxygenase, cytochrome-P450 and reactive oxygen species-triggered reactions [16]. While many products of these enzymes and of the nonenzymatic oxidation of AA elicit a variety of cellular responses acting via specific receptors, unmetabolized AA also possesses biological activity on its own. As a result of its structural features, with four methylene-interrupted *cis* double bonds, AA influences cellular membrane fluidity thereby regulating the function of key membrane proteins involved in signal transduction [56,57]. In the current study, we extend our previous observations on the role of AA in inducing a foamy phenotype in human monocytes and provide further evidence that this is an effect mediated by the fatty acid itself and not by an oxygenated metabolite.

This work also highlights a striking difference between normal monocytes and lipid-laden cells, i.e., the presence of elevated amounts of AA in the TAG fraction of foamy monocytes, which does not occur in normal circulating monocytes. In the latter cells, AA is only present in membrane phospholipids. Thus, the key question arises as to whether the AA accumulating in neutral lipids can be mobilized during inflammatory activation of the monocytes and then further utilized to generate bioactive lipid mediators. This is particularly relevant because AA-laden monocytes may migrate to sites of inflammation, where they could potentially discharge their AA reservoirs in response to local proinflammatory stimuli, thereby exacerbating damage. Moreover, the existence of alternative pathways for AA release within the same cell that depend on substrate usage (i.e., phospholipid versus TAG), would also confer on the system a greater versatility in terms of cell regulation of the lipid-mediated inflammatory response (i.e., phospholipase regulation versus ATGL regulation).

To address the aforementioned questions, in-depth mass spectrometry-based lipidomic analyses combined with strategies to obtain monocytes with a variable cell AA content have been carried out in this study. A correspondence was found between exposing the cells to varied amounts of AA and the appearance of the fatty acid in TAG, but not in membrane phospholipids. This is consistent with the notion that TAG may constitute an expandable pool for accommodating excess AA that cannot enter the higher affinity, lower capacity phospholipid pool [17,42,61]. However, we have failed to appreciate any correspondence between the amount of AA in TAG and the capacity of the cells to increase their AA mobilization response, which remained essentially the same, regardless of how much AA was present in TAG. Even under conditions where TAG constituted the major cellular AA store, no significant TAG hydrolysis was detected, and the AA release response was entirely attributable to phospholipid hydrolysis. Analysis of the phospholipid classes involved in the process implicate all three major AA-containing phospholipid classes, namely PC, PE and PI, which is in general agreement with previous data [33].

The lack of mobilization of AA from the neutral lipid pool is striking, especially considering that LDs have been amply suggested to act as an intracellular site for eicosanoid biosynthesis, and that major enzymes of the AA cascade such as cPLA_2_α and cyclooxygenase-2 associate with this organelle [2,3]. While a direct demonstration that cPLA_2_α cleaves phospholipids to release AA from the LD surface monolayer is yet to be provided [62], the enzyme is known to be essential for maintaining the LD structure, in a process that may be related to lysophospholipid formation [3]. Since AA mobilization in innate immune cells involves cross-talk between cPLA_2_α and certain secreted phospholipase A_2_ forms [63], and the latter enzymes are known to also act intracellularly [64,65,66,67], it is possible that not only cPLA_2_α but also a secreted phospholipase A_2_ acts to hydrolyze phospholipids at the LD monolayer, as it occurs with serum lipoproteins, which are structurally similar to LDs [68].

Once phospholipid hydrolysis occurs at the LD surface, the fatty acid pool must be preserved in order to maintain the LD integrity. Thus, recycling of fatty acids between the LD surface phospholipids and neutral lipids from the inner core could occur to maintain homeostasis [62]. Whether this fatty recycling involves AA under activation conditions is unknown at present, but cells of the innate immunity possesses all the enzyme machinery necessary to transfer AA from TAG to phospholipids via CoA-dependent reactions [69]. In this regard, it could be argued that the opposite set of reactions, i.e., the transfer of AA from phospholipids to TAG to replenish the AA that could potentially be lost from this pool by direct release, could also be possible. This would result in a net release of AA from TAG in the absence of apparent changes in mass levels of AA-containing TAG. Such a situation is somewhat reminiscent of what has been observed when murine peritoneal macrophages are stimulated with unopsonized zymosan to release AA [35,38,59]. In this system, PE levels appear to change little after cellular stimulation, because almost all of the AA removed by cPLA_2_α is replenished by CoA-independent transacylase, which transfers AA from diacyl-PC to diacyl-PE and alkenyl-PE. However, the above scenario appears unlikely because our experiments using BEL, a potent inhibitor of patatin-like phospholipases such as ATGL [51,52,53,70], had no measurable effect on the AA mobilization from activated foamy monocytes.

In conclusion, our results demonstrate that membrane phospholipids are the major, if not the only, sources for the AA mobilization from stimulated human monocytes, even under conditions where cellular levels of the fatty acid in TAG exceed those in membrane phospholipids. Our results, in the context of the existing literature, are thus fully consistent with work by Johnson et al. [71] in human neutrophils and differ from those of Dichlberger et al. [72] in human mast cells. In the latter study, silencing of ATGL in mast cells reduced the synthesis of eicosanoids generated by both cyclooxygenase and lipoxygenase pathways to the same extent as that observed with cPLA_2_α silencing [72]. As discussed elsewhere [73], apparently the pathways responsible for AA release may differ in a tissue- and cell-specific manner. Possible pathophysiological implications and/or biological reasons for the presence of AA in TAG in monocytes, if any, remain to be elucidated. Given that high levels of free AA are toxic to cells [10,55,56,57], one intriguing possibility to consider is that the AA entry into TAG may serve as an expandable storage pool to protect the cells from the toxicity of the fatty acid or to limit inflammation by reducing the amount of free fatty acid available for eicosanoid synthesis.

## Figures and Tables

**Table 1:** Products of AA detected in commercial samples of the fatty acid.

Eicosanoid	LC Retention time (min)	[M−H]^−^ (m/z) ^a^	Product Ion (m/z) ^b^
15-oxoETE	14.30	317	219
12-oxoETE	14.91	317	153
5-oxoETE	15.78	317	203
15-HETE	13.92	319	175, 219
11-HETE	14.56	319	167
12-HETE	14.93	319	179, 208
8-HETE	15.01	319	155, 163
9-HETE	15.15	319	151
5-HETE	15.71	319	115
14,15-EET	15.75	319	219
11,12-EET	16.28	319	167, 179
8,9-EET	16.31	319	155, 167
5,6-EET	16.49	319	191
15-HPETE	14.30	335	113
12-HPETE	15.35	335	153
5-HPETE	16.53	335	155

^a^ Carboxylate anions by negative ion electrospray ionization. ^b^ Specific product ions for experiments using tandem mass spectrometry in product-ion scan mode are indicated. HETE, hydroxyeicosatetraenoic acid; EET, epoxyeicosatrienoic acid; HPETE, hydroperoxy- eicosatetraenoic acid; oxoETE, oxoeicosatetraenoic acid.

## Abbreviations

AA	arachidonic acid
ATGL	triacylglycerol lipase
CE	cholesteryl esters
DAG	diacylglycerol
GC/MS	gas chromatography coupled to mass spectrometry
LC/MS	liquid chromatography coupled to mass spectrometry
LD	lipid droplet
MAG	monoacylglycerol
cPLA_2_α	group IVA cytosolic phospholipase A_2_α
PL	phospholipids
PC	choline-containing phospholipids
PE	ethanolamine-containing phospholipids
PI	phosphatidylinositol
PS	phosphatidylserine
TAG	triacylglycerol
